# Supplementary material for: Optimal percolation on multiplex networks
Source: Nat Commun. 2017 Nov 16;8:1540. doi: 10.1038/s41467-017-01442-2 (PMC5691044; doi:10.1038/s41467-017-01442-2)
Supplement: Supplementary file 1 — Supplementary Information [file 41467_2017_1442_MOESM1_ESM.pdf]

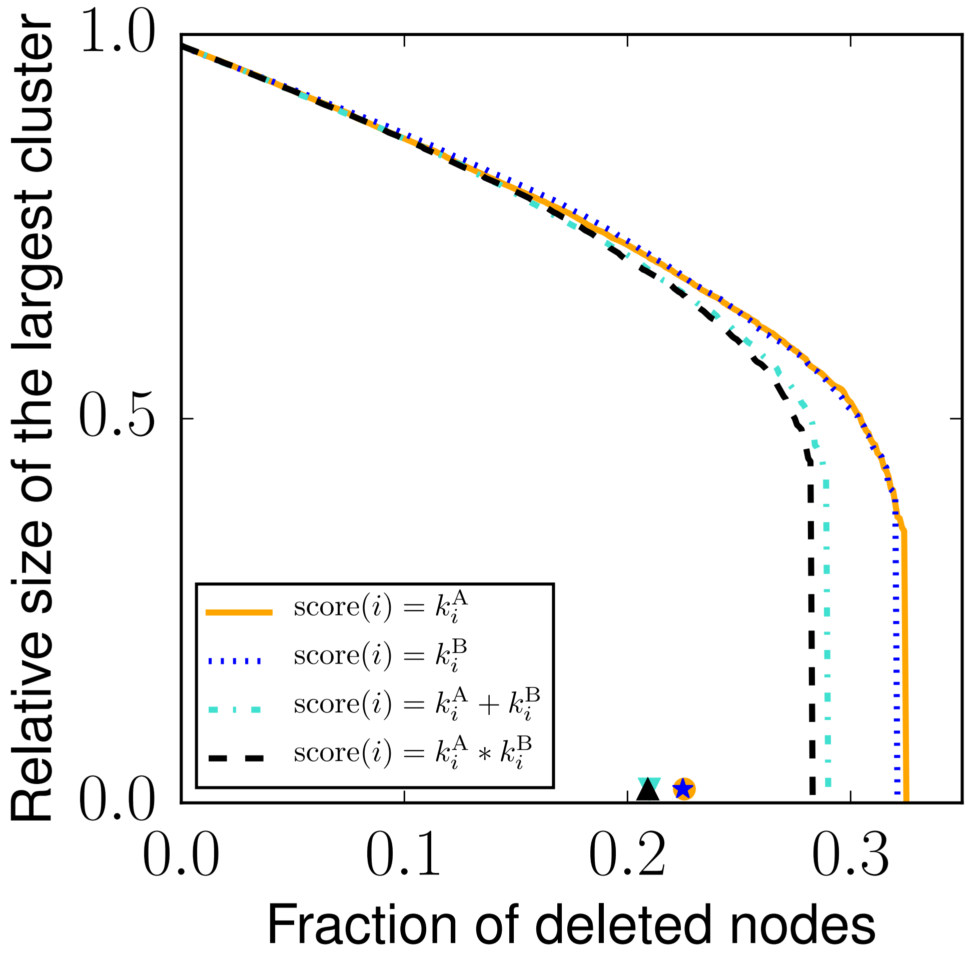

Supplementary Figure 1. Optimal percolation for a multiplex network using the High Degree (HD) algorithm. We consider a multiplex network composed of two identical layers with $N = 10000$ nodes generated according to the Erdős–Rényi model with average degree $\langle k \rangle = 5.0$. Different line styles correspond to four different methods of defining node scores in multiplex networks. Markers are the dismantling fraction obtained with these four methods when combined with the Greedy Reinserting (GR) procedure (see Supplementary Note 2).

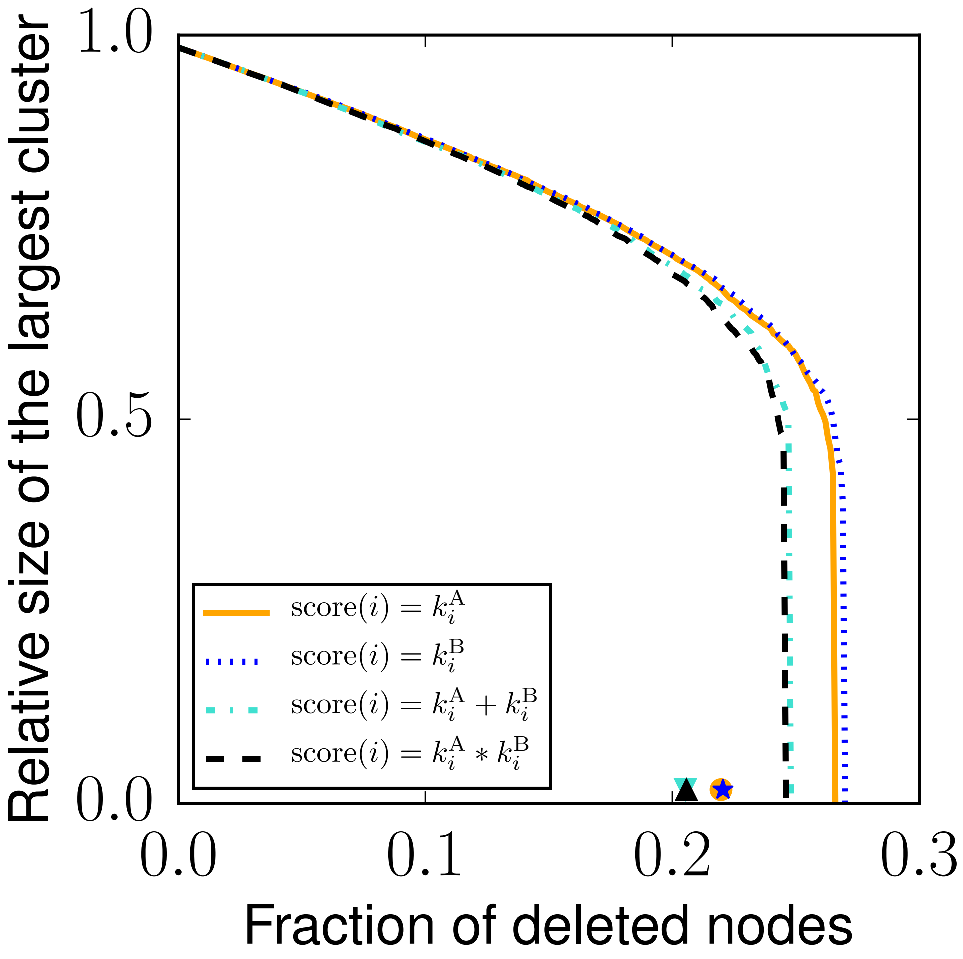

Supplementary Figure 2. Optimal percolation for a multiplex network using the High Degree Adaptive (HDA) algorithm. We consider a multiplex network composed of two identical layers with $N = 10000$ nodes generated according to the Erdős–Rényi model with average degree $\langle k \rangle = 5.0$. Different line styles correspond to four different methods of defining node scores in multiplex networks. Markers are the dismantling fraction obtained with these four methods when combined with the Greedy Reinserting (GR) procedure (see Supplementary Note 2).

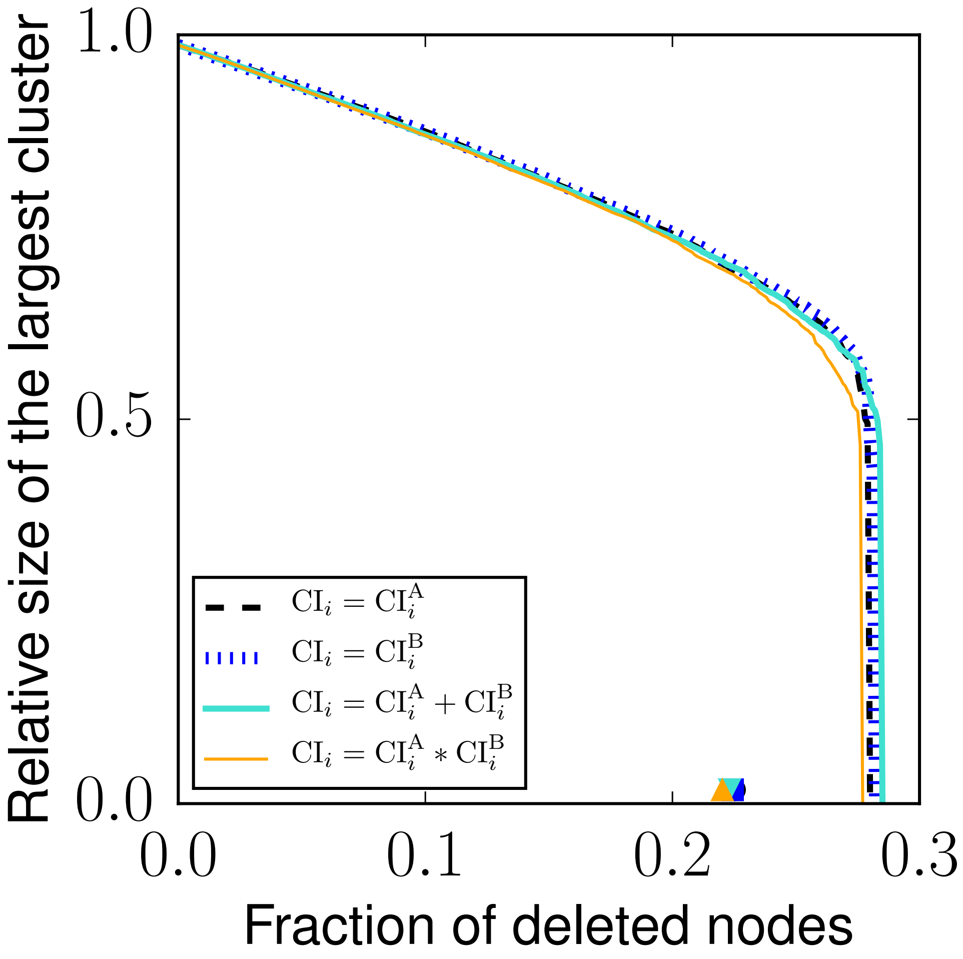

Supplementary Figure 3. Optimal percolation for a multiplex network using the Collective Influence (CI) algorithm. We consider a multiplex network composed of two identical layers with $N = 10000$ nodes generated according to the Erdős–Rényi model with average degree $\langle k \rangle = 5.0$. Different line styles correspond to four different methods of defining CI scores in multiplex networks. Markers are the dismantling fraction obtained with these four methods when combined with the Greedy Reinserting (GR) procedure (see Supplementary Note 2).

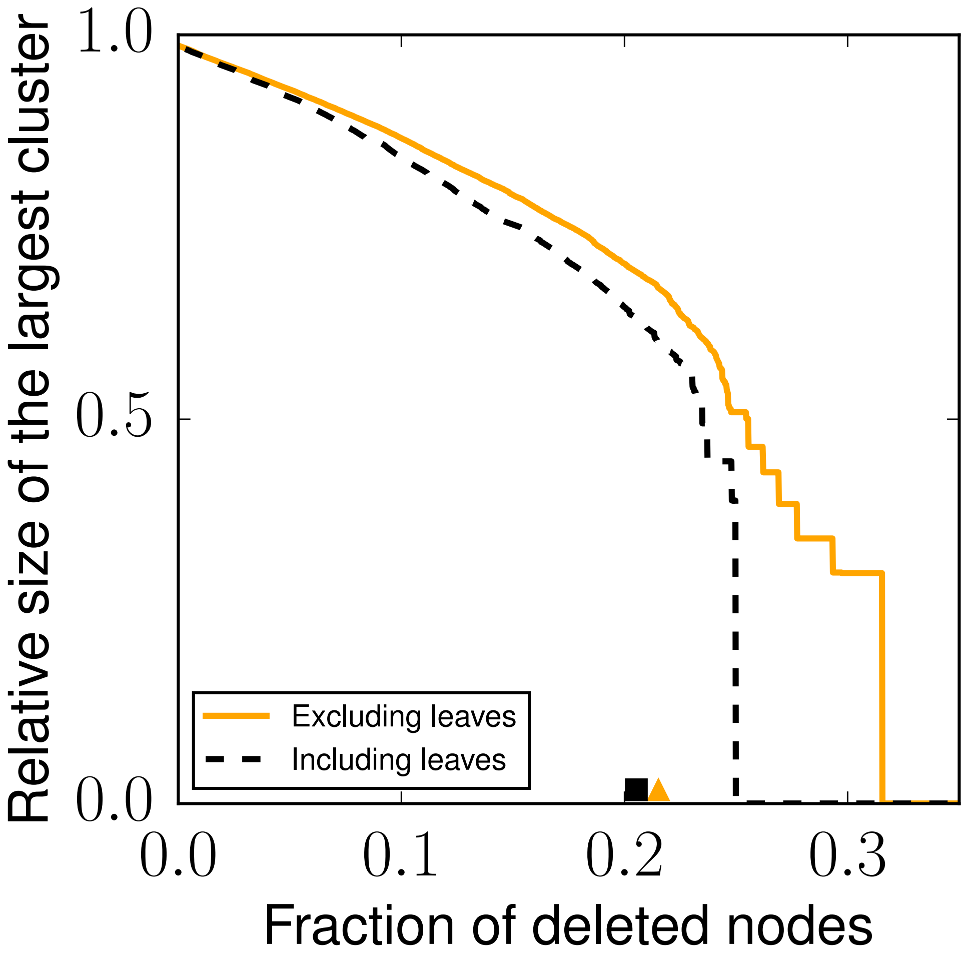

Supplementary Figure 4. Optimal percolation for a multiplex network using the Explosive Immunization (EI) algorithm. We consider a multiplex network composed of two identical layers with $N = 10000$ nodes generated according to the Erdős–Rényi model with average degree $\langle k \rangle = 5.0$. Different line styles correspond to two different kernels: Eq. 3 that does not exclude the leaves and Eq. 3 which excludes the effect of leaves by replacing $\sqrt{|M|}$ with $\sqrt{|M|} - 1$. Markers are the dismantling fraction obtained with these two methods when combined with the Greedy Reinserting (GR) procedure (see Supplementary Note 2).

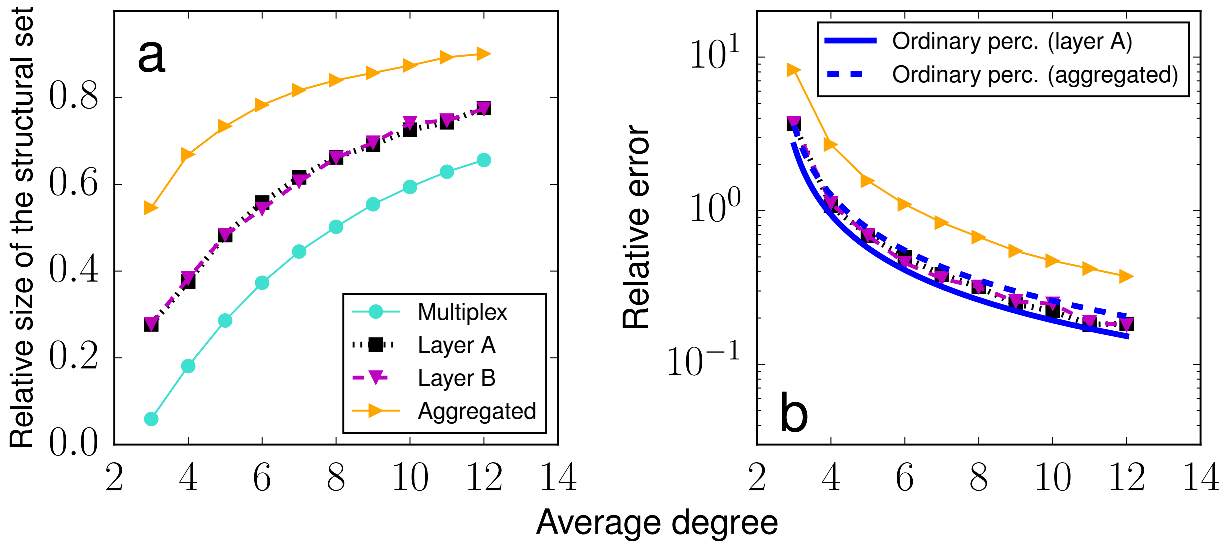

Supplementary Figure 5. Optimal percolation problem in synthetic multiplex networks using HD algorithm. a) We consider multiplex networks with $\mathrm{N} = 10^5$ and layers generated independently according to the Erdős–Rényi model with average degree $\langle k \rangle$. We estimate the relative size of the set of SNs on the multiplex as a function of $\langle k \rangle$ (turquoise circles), and compare it with the same quantity but estimated on the individual layers (black squares and purple triangles) or the aggregated (orange triangles). b) The relative errors of single-layer estimates of the size of the structural set with respect to the ground-truth value provided by the multiplex estimate. Colors and symbols are the same as those used in panel (a). The blue curves with no markers represent instead the theoretically expected behaviour for an ordinary percolation process.

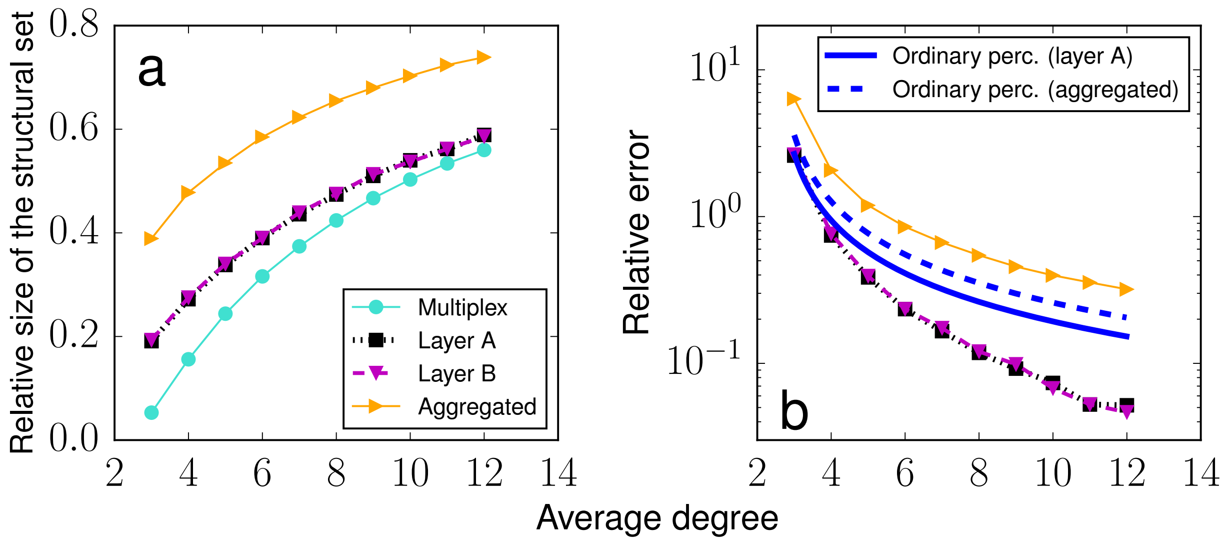

Supplementary Figure 6. Optimal percolation problem in synthetic multiplex networks using HDA algorithm. a) We consider multiplex networks with $N = 10^5$ and layers generated independently according to the Erdős–Rényi model with average degree $\langle k \rangle$. We estimate the relative size of the set of SNs on the multiplex as a function of $\langle k \rangle$ (turquoise circles), and compare it with the same quantity but estimated on the individual layers (black squares and purple triangles) or the aggregated (orange triangles). b) The relative errors of single-layer estimates of the size of the structural set with respect to the ground-truth value provided by the multiplex estimate. Colors and symbols are the same as those used in panel (a). The blue curves with no markers represent instead the theoretically expected behaviour for an ordinary percolation process.

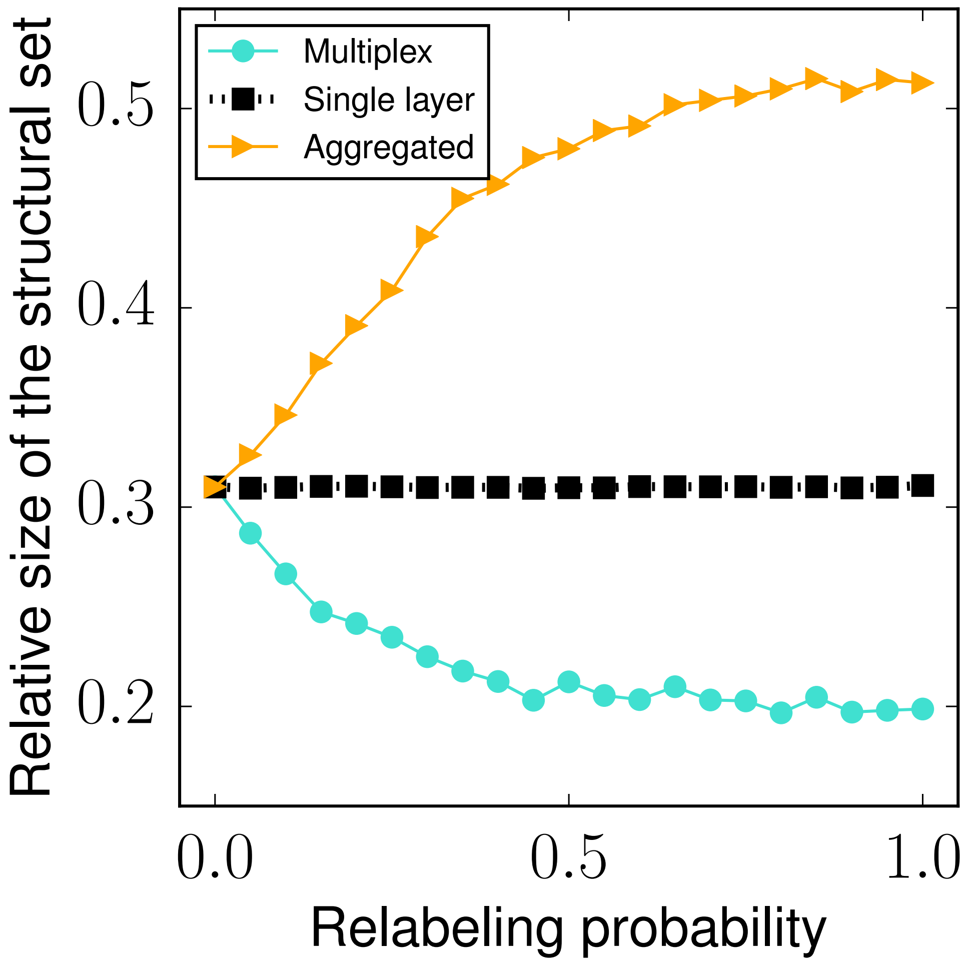

Supplementary Figure 7. The effect of reducing edge overlaps and interlayer degree-degree correlation by partially relabeling nodes in multiplex networks with initially identical layers. Initially, both layers are a copy of a random network generated by an Erdős-Rényi model with $N = 1,000$ nodes and average degree $\langle k \rangle = 5$. Then, in one of the layers, each node is selected to switch its label with another randomly chosen node with a certain probability α. For each α, we determine the mean of the relative size of the set of SNs over 100 realizations of the HDA+GR algorithm on the multiplex network.

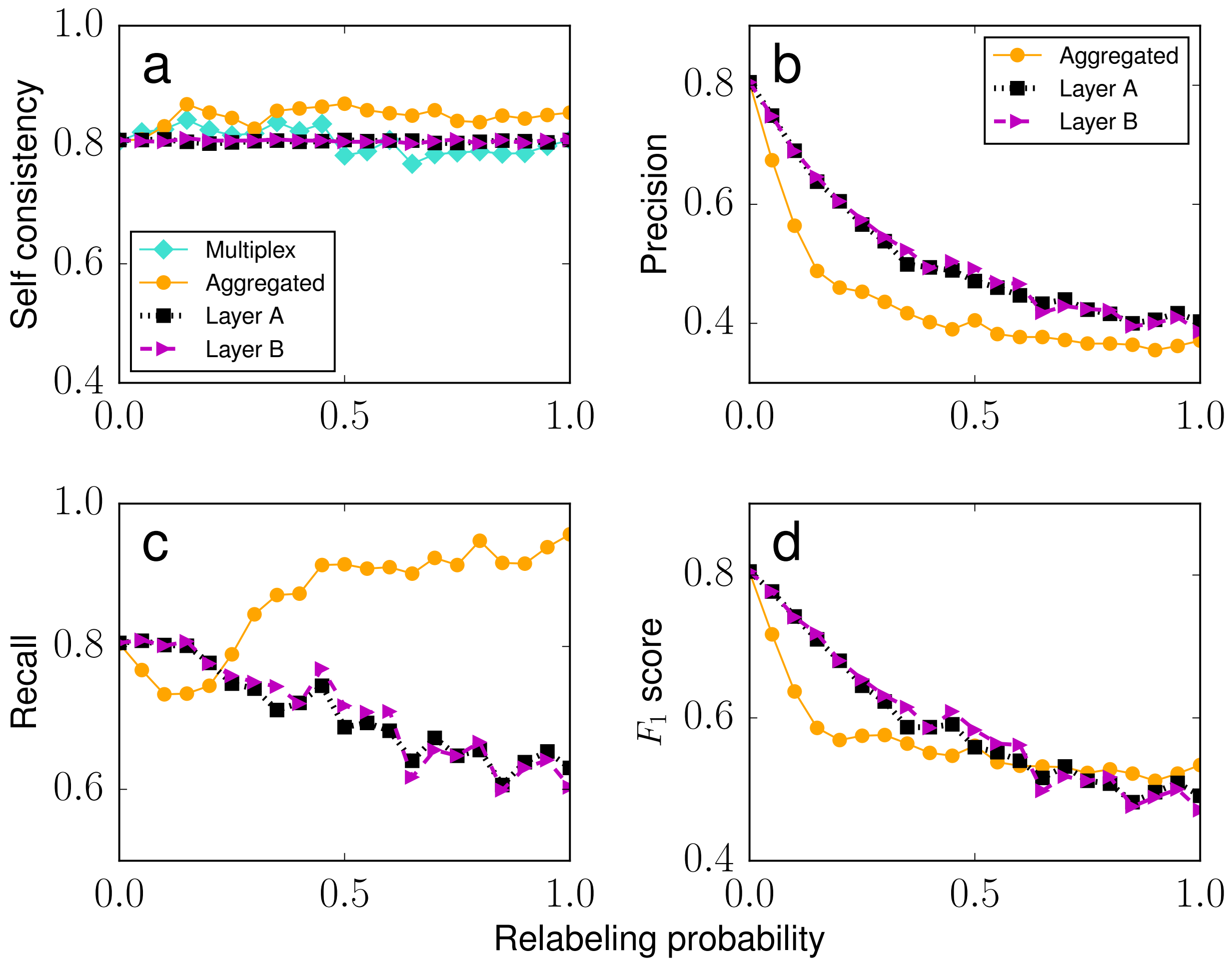

Supplementary Figure 8. The effect of reducing edge overlaps and interlayer degree-degree correlation by partially relabeling nodes in multiplex networks with initially identical layers. We consider the multiplex networks described in Supplementary Figure 7 and the sets of SNs found for the multiplex and single layer based representations of these networks. a) As the set of SNs found in different instances of the optimization algorithm are different from each other, we first quantify the self-consistency of those solutions across 100 independent runs of the HDA+GR algorithm. We then assume that the multiplex representation provides the ground-truth classification of the nodes. We compare the results of the other representation with the ground truth by measuring their precision (panel b), their sensitivity or recall (panel c), and their F_1 score (panel d).

Supplementary Note 1. DISMANTLING ALGORITHMS

In this section, we briefly discuss some of the most effective dismantling algorithms on monoplex networks and their generalization to multiplex networks with two layers. The aim of these algorithms is to approximate the set of structural nodes which is the minimal set of nodes that their removal dismantles the network into vanishingly small (non-extensive) clusters. We first introduce some of the score-based algorithms. In such algorithms, at each step, a score for each node is calculated and the node with the highest score is removed (when several nodes have the same score, one of them is removed at random). We discuss four different types of such algorithms: High Degree (HD), High Degree Adaptive (HDA), Collective Influence (CI) and Explosive Immunization (EI). These methods are partially deterministic in nature, i.e., nearly the same set of structural nodes are discovered at each realizations of the algorithm. Besides score-based algorithms, we present Simulated Annealing (SA) algorithm which is a greedy algorithm that searches the solution space of the dismantling problem to find the best approximation to the structural sets. The SA method takes into account the collective behavior of the dismantling problem and provides several dismantling sets for different realizations of the algorithm on the same network structure.

High Degree (HD)

In a monoplex network, the easiest way to dismantle a network, is a degree-based attack. After sorting the nodes with respect to their degrees, the nodes with the highest degree are removed one by one (in cases where there are more than one node with a certain degree, one of them is removed at random), until the network is dismantled. As this algorithm is deterministic (except from the randomness in choosing a node from those with the same degree), the set of nodes that are removed to dismantle the network is almost unique.

In multiplex networks, the degree of a node can be defined in various ways. We consider four different cases: the score of a node is defined as (i) its degree in layer A, (ii) its degree in layer B, (iii) the sum of its degrees across all the layers, and (iv) the product of its degrees across all the layers. It is worth mentioning that, when using HD, HDA and CI methods, at each step we remove $0.001 \times N$ of the nodes, where N is the total number of nodes (in each layer) of the multiplex.

As Supplementary Figure 1 illustrates, to destruct a multiplex, the two scores defined as a combination of degrees in different layers are more effective than those based on the degrees in only one of the layers. In the main script and in the rest of the Supplemental Material (SM) when we refer to HD method, we mean the one in which the score of a node is the product of its degrees across all the layers.

High Degree Adaptive (HDA)

In the HD algorithm if we take into account the history of the process and recalculate, at each step, the degrees of the nodes, it is referred to as an HDA algorithm. Since the HDA algorithm is adaptive it is expected to work better than the HD method. In each step of the monoplex version of the HDA, we remove a fraction $0.001 \times N$ of the nodes that had the highest degrees; then we recalculate the degree of the nodes present in the Giant Connected Component (GCC) of the network. We repeat this process until the size of the GCC reduces

to $\sqrt{N}$ or smaller; this threshold satisfies the condition that in the dismantled network the size of all the clusters is a sub-linear function of N.

Like the HD case, we can define at least four methods to define the degree of a node. Please notice that in the multiplex version, when we update the degrees, we exclude those neighbors that are not in the Giant Mutually Connected Component (GMCC) of the network. Supplementary Figure 2 shows the effectiveness of the HDA algorithm for the different definitions of nodes' degrees. Similar to the results for HD, it is more effective to combine the scores of different layers, than considering layers as isolated networks. In all the subsequent sections and in the main script, when we refer to HDA, we mean the one in which the score of a node is defined as the multiplication of its degrees across all the layers.

Collective Influence (CI)

In the monoplex version of the CI algorithm [1], the score $\mathrm{CI}_i(l)$ of node i is equal to the excess degree of i multiplied by the sum of the excess degrees of its neighbours at a specific distance l from i:

$$\mathrm{CI}_i(l) = (k_i - 1) \sum_{j \in \partial \mathrm{Ball}(i,l)} (k_j - 1), \tag{1}$$

where $\partial\mathrm{Ball}(i, l)$ denotes the neighbors of i at the distance l (i.e., the nodes that have a geodesic distance l from i). At each step, the CI score is adaptively calculated for all the nodes; then nodes with the highest score are removed from the network, until the network is dismantled. It was shown [1] that the performance of the CI method increases with l up to $l = 4$; for $l > 4$ the performance is not improved appreciably as l is increased.

To adapt the CI algorithm to multiplex networks with two layers, we considered several possible definitions of the CI score in the multiplex: (i) using the CI obtained based only on the structure of layer A, (ii) based only on the structure of layer B, (iii) the sum of the CIs of a node in layer A and layer B, and (iv) the product of these two CI scores. Supplementary Figure 3 illustrates that, the generalizations of the CI method we considered here are not as effective as those derived based on the HD (Supplementary Figure 1) and HDA (Supplementary Figure 2) methods. Thus, methods based on the CI measures of the layers do not provide an effective algorithm for the optimal percolation problem.

Explosive Immunization (EI)

The EI algorithm is based on a method referred to as explosive percolation. The original explosive percolation method was introduced by Achlioptas *et al.* [2]. In this method at first all the edges are removed; then they are gradually reintroduced to the network, but in a specific order that prevents the formation of the GCC, until a point where the formation of the GCC is inevitable. To add a new edge, first several random edges are selected. Then a score is calculated for each of the selected edges using a predefined kernel (a possible kernel, for example, defines the score as the sum of the sizes of the two clusters connected by the corresponding edge). Then the edge with the minimum score is added back the network. The scores represent the contribution of each edge in the formation of the giant cluster. When the network reaches the point where the formation of the giant cluster is inevitable, the rest of the edges are added back using the same kernel.

A problem related to explosive percolation is the optimal immunization [3] in which the goal is to find the blocker nodes, which if get vaccinated, the giant connected component of the susceptible nodes breaks down; this break down eliminates a large scale epidemic spread. Clusella *et al.* [3] proposed a reverse approach to find the blockers. They introduced an algorithm that locates instead all the nodes that are irrelevant to the formation of the giant susceptible cluster. In this respect, their algorithm is a modified version of the explosive percolation. Their algorithm considers the site percolation version of the explosive percolation, in which all the links are present but, in the beginning, all the nodes are absent. Then, all the non-blocker nodes (that have no contribution to the formation of the giant susceptible cluster) are added gradually. The remaining nodes are the blocker nodes which should be vaccinated. We refer to this method as the explosive immunization (EI) algorithm.

For a monoplex network we implement the EI algorithm as follows. At each step, we select $N^{(\mathrm{C})} = 1000$ candidate nodes from the set of absent nodes, and calculate the score σ_i of each of them using the following kernel:

$$\sigma_i = \sum_{j \subset N_i} \left(\sqrt{|C_j|} - 1 \right) + k_i^{(\mathrm{eff})}, \tag{2}$$

where, N_i represents the set of all connected components (CCs) linked to node i, each of which has a size C_j, and ${k_i}^{(\mathrm{eff})}$ is an effective degree attributed to each node (please see Ref. [3] for the details). Then the nodes with the lowest scores are added to the network. This procedure is continued until the size of the GCC exceeds a predefined threshold g^* (For the simulations of this paper we used $g^* = \sqrt{N}$). The minus one term in Eq. 2 is excluding any leaves connected to node i, since they do not contribute to the formation of the GCC and should be ignored in the score of a node.

In our extension of the EI method to multiplex networks, we consider the different kernel but otherwise perform the exact same procedure as the one described above. The new kernel (Eq. 3) we use is based on the sizes of the mutually connected components (MCCs) rather than on the sizes of CCs:

$$\sigma_i = 1/2 \left[\sum_{j \subset N_i^{[\mathrm{A}]}} \left(\sqrt{|M_j|} \right) + \sum_{j \subset N_i^{[\mathrm{B}]}} \left(\sqrt{|M_j|} \right) \right] + \sqrt{k_i^{[\mathrm{A}](\mathrm{eff})} k_i^{[\mathrm{B}](\mathrm{eff})}}, \tag{3}$$

where $N_i^{[\mathrm{A}]}$ is the set of neighbors of node i in layer A, M_j is the size of the MCC to which node j belongs, and $k_i^{[\mathrm{A}](\mathrm{eff})}$ is the effective degree of i in layer A obtained using the same definition proposed [3] for the monoplex version of the EI method.

In Eq. 3 we do not add a minus 1 term to exclude the leaves; the reason is that while in monoplex networks leaves do not have a significant contribution in the formation of the GCC, in multiplex networks even a leaf node is important in the formation of the GMCC. This is because at the sub-critical regime of multiplex networks usually most of the MCCs are isolated nodes or have very small sizes. Supplementary Figure 4 certifies that if the leaves were excluded instead, the performance of the algorithm would decrease.It is worth to mention that in the simulations of Figure 1 of the main text, we used a $N^{(\mathrm{C})} = 1000$.

Simulated Annealing (SA)

The simulated annealing (SA) method has been used for the dismantling problem in monoplex networks [4]. Generally an SA algorithm defines an energy function that attributes

energy values to each configuration of the system. The phase space of the system is searched for the optimal configuration (the one with the minimum energy) by Markov Chain Monte Carlo moves that switch the system from one configuration to another. In dismantling of multiplex networks, the algorithm should find the minimal set of nodes which if deleted the size of the GMCC becomes non-extensive. Each configuration of the multiplex network is represented by $\{R, g\}$, where R and g are, respectively, the number of removed nodes (each node and all its corresponding replica nodes are counted as one node), and the relative size of the GMCC. The energy of a configuration is defined as follows:

$$\varepsilon = Rv + g, \tag{4}$$

where v is the cost of removing a node from the multiplex network and in the simulations presented in this paper it is set $v = 0.6$. At each step t of the algorithm, one node, present or removed, is selected at random; then one of the following sets of operations are performed:

- If the node is present and it belongs to the GMCC, it is removed (thus $R_t = R_{t-1} + 1$) and the new size of the GMCC (g_t) is calculated.
- If the node is present but it does not belong to the GMCC, then it is removed (thus $R_t = R_{t-1} + 1$); but since it did not belong to the GMCC, $g_t = g_{t-1}$.
- If the node is in the set of removed nodes, it is added back to the network and $R_t = R_{t-1} - 1$. Then M_i (the size of the MCC formed after inserting i) is calculated and $g_t = \max(M_i, g_{t-1})$.

Afterwards the energy of the new configuration ε_t is calculated and the set of operations is accepted with a probability equal to $\min\left(1, \mathrm{e}^{-\beta(\varepsilon_{\text{new}} - \varepsilon)}\right)$. If it is accepted, the new configuration ($\{R_t, g_t\}$) is retained, otherwise, the operations are omitted and the old configuration ($\{R_{t-1}, g_{t-1}\}$) is preserved.

Here, β is interpreted as the inverse of the temperature of the annealing process. The SA algorithm starts with a $\beta_{\min}$ and, at each step, β is slightly increased by $\delta\beta$. A smaller $\delta\beta$ means a slower decrease in the temperature which allows the SA method to better search for the optimal configurations, at the expense of increasing the running time of the algorithm. In this paper we change the values of β from 0.5 to 20.0 with $\delta\beta = 10^{-6}$.

In Figure 1 of the main text, we show that the SA method outperforms the four score-based algorithms; thus, for the analysis of the optimal percolation problem, we mostly use the SA method (see the main text). In Supplementary Note 3, we also provide results for the second best algorithm, i.e., the HDA method and show that the results are qualitatively similar to those of the SA method.

Supplementary Note 2. GREEDY REINSERTING (GR)

After a network (either isolated or multiplex) is dismantled using a greedy or score-based algorithm, there are some removed nodes that if added back to the network, the size of the GMCC is not increased substantially, i.e., no cluster with an extensive size is created if they are reinserted. Such nodes may have been removed because the greedy or score-based algorithms are not exact in the sense that they do not take into account the collective nature of the dismantling problem. An approach that addresses this issue is referred to as the greedy reinserting (GR) method [4, 5]. In the GR method, after a set of structural nodes is detected using another algorithm, at each step a randomly chosen node from the set is reinserted to the network, and unless its reinsertion does not increase the size of the GMCC to a threshold $\sqrt{N}$, it is removed again. This process is continued until practically none of the nodes remained in the set can be added to the network without keeping the size of the GMCC non-extensive.

As shown in Supplementary Figures 1-4 and Figure 1 of the main text, the GR method boosts effectively the performance of every one of the score-based dismantling algorithms and returns sets of structural nodes with almost identical sizes irrespective of the initial algorithm used. Moreover, the result of each of the score-based algorithms combined with the GR method is nearly as good as that of the SA method (the SA method itself is not improved appreciably by applying a GR method afterwards). These results suggest that probably the sets obtained by the SA method and any one of the score-based algorithms combined with GR are to a considerable extent similar to each other.

Supplementary Note 3. COMPLEMENTARY RESULTS FOR THE DEGREE-BASED METHODS

In this section, we provide further results for the HD (Supplementary Figure 5) and the HDA (Supplementary Figure 6) methods, and also for the combination of the GR method with HDA (Supplementary Figures 7–8); we compare these results with some of the results of the SA algorithm presented in the main text. In contrast to the SA algorithm, the degree-based algorithms are much more efficient in terms of the running time; hence, we were able to produce some of the results (see Supplementary Figures 5–6) for larger ER networks.

Supplementary Figures 5 and 6 show that, for both HD and HDA performed on the aggregated representation, the behavior of q_c (the relative size of the set of structural nodes) with respect to the network average degree resembles the results of the SA algorithm. On the other hand, HDA matches better to the result of SA for optimal percolation on each of the single layers of the multiplex network. In particular, for networks with sufficiently large degree, HDA on each of the layers can find a q_c very close to the q_c it obtains for the multiplex representation.

As shown in Supplementary Figure 7, the behavior of q_c with respect to the relabeling probability (higher relabeling probability indicates lower density of overlapping edges and lower interlayer degree-degree correlation) obtained with the HDA+GR method is qualitatively similar to the results of the SA algorithm (Figure 3 of the main text). Moreover, Supplementary Figure 8a shows that, as expected, HDA+GR has a relatively higher self-consistency compared to that of the SA algorithm reported in Figure 4 of the main text. It is worth noting that, in contrast to SA, the self-consistency of HDA+GR does not decrease with the relabeling probability in the multiplex representation (Supplementary Figure 8a).

Interestingly, despite the qualitative similarity of the results, HDA+GR returns higher values of precision (Supplementary Figure 8b), recall (Supplementary Figure 8c), and F_1-score (Supplementary Figure 8d) than those of SA (see Figure 4 of the main text). As there is not much randomness in HDA+GR, the structural nodes are dominantly determined by the sequence of (adaptive) degrees of the nodes and the sets from different network representations have a higher overlap compared to those find by the SA algorithm.

SUPPLEMENTARY REFERENCES

[1] Morone, F. & Makse, H. A. Influence maximization in complex networks through optimal percolation. *Nature* **524**, 65–68 (2015).

[2] Achlioptas, D., D'souza, R. M. & Spencer, J. Explosive percolation in random networks. *Science* **323**, 1453–1455 (2009).

[3] Clusella, P., Grassberger, P., Pérez-Reche, F. J. & Politi, A. Immunization and targeted destruction of networks using explosive percolation. *Phys. Rev. Lett.* **117**, 208301 (2016).

[4] Braunstein, A., DallAsta, L., Semerjian, G. & Zdeborov, L. Network dismantling. *Proc. Natl. Acad. Sci. USA* **113**, 12368–12373 (2016).

[5] Zdeborová, L., Zhang, P. & Zhou, H.-J. Fast and simple decycling and dismantling of networks. *Sci. Rep.* **6**, 37954 (2016).
